# Aesthetic Surgery Advice and Counseling from Artificial Intelligence: A Rhinoplasty Consultation with ChatGPT

**DOI:** 10.1007/s00266-023-03338-7

**Published:** 2023-04-24

**Authors:** Yi Xie, Ishith Seth, David J. Hunter-Smith, Warren M. Rozen, Richard Ross, Matthew Lee

**Affiliations:** 1 Department of Plastic Surgery, Peninsula Health, Melbourne, Victoria 3199 Australia; 2 Faculty of Medicine, Monash University, Melbourne, Victoria 3004 Australia

**Keywords:** ChatGPT, Artificial intelligence, Chatbot, Rhinoplasty

## Abstract

**Background:**

ChatGPT is an open-source artificial large language model that uses deep learning to produce human-like text dialogue. This observational study evaluated the ability of ChatGPT to provide informative and accurate responses to a set of hypothetical questions designed to simulate an initial consultation about rhinoplasty.

**Methods:**

Nine questions were prompted to ChatGPT on rhinoplasty. The questions were sourced from a checklist published by the American Society of Plastic Surgeons, and the responses were assessed for accessibility, informativeness, and accuracy by Specialist Plastic Surgeons with extensive experience in rhinoplasty.

**Results:**

ChatGPT was able to provide coherent and easily comprehensible answers to the questions posed, demonstrating its understanding of natural language in a health-specific context. The responses emphasized the importance of an individualized approach, particularly in aesthetic plastic surgery. However, the study also highlighted ChatGPT’s limitations in providing more detailed or personalized advice.

**Conclusion:**

Overall, the results suggest that ChatGPT has the potential to provide valuable information to patients in a medical context, particularly in situations where patients may be hesitant to seek advice from medical professionals or where access to medical advice is limited. However, further research is needed to determine the scope and limitations of AI language models in this domain and to assess the potential benefits and risks associated with their use.

**Level of Evidence V:**

Observational study under respected authorities. This journal requires that authors assign a level of evidence to each article. For a full description of these Evidence-Based Medicine ratings, please refer to the Table of Contents or the online Instructions to Authors www.springer.com/00266.

## Introduction

The human nose is a significant facial feature that plays a crucial role in facial aesthetics and identity. [1, 2] Rhinoplasty is a complex surgical procedure with historical significance. It is aimed at improving both the functional and aesthetic aspects of the nose following trauma or disease-related deformities. [3] The objective of aesthetic rhinoplasty is to create a natural-looking nose that integrates well with the rest of the face, with no visible signs of surgical intervention, and that allows the patient to breathe freely. [4] Being one of the most sought after surgeries globally, patients often have numerous questions regarding the procedure, which they may feel too self-conscious to ask or may not even be aware they need to inquire about.

Artificial intelligence (AI) language-generated tools present a promising avenue to revolutionize the delivery of scientific information. One such tool is ChatGPT, a large language model capable of generating human-like text which has attracted significant attention for its potential to assist researchers in writing scientific papers and performing literature reviews. Trained on massive amounts of text data from a wide variety of sources on the internet, ChatGPT is capable of providing logical, comprehensible and accurate responses to almost any question, including those of a medical nature.

Despite the rapid expansion of AI, there is still limited understanding of its potential value for public inquiry. To address this gap, the authors conducted a hypothetical rhinoplasty consultation, using ChatGPT to generate answers to questions commonly asked by patients, and evaluating its responses. The authors propose that the integration of AI and language models, such as ChatGPT, in medical consultation holds significant promise for improving patient education and satisfaction. As AI and machine learning continue to advance, they may enable novel approaches to enhancing patient outcomes.

## Methods

### Aim

In this study, we aimed to investigate the potential of artificial intelligence language models to serve as clinical assistants. For this purpose, we employed ChatGPT, one of the largest language models currently accessible to the public, and evaluated its capacity, effectiveness, and accuracy in providing perioperative information to a patient.

### Study Design

We asked ChatGPT nine hypothetical questions simulating a doctor-patient consultation for rhinoplasty surgery. The questions were based on a comprehensive rhinoplasty consultation checklist published on the American Society of Plastic Surgeons website [5]. The scenario objective was to cover a broad cross section of information that a prospective patient might want to know. The responses provided by ChatGPT were assessed for accuracy, informativeness, and accessibility by Specialist Plastic Surgeons (DHS, WR, RR and ML) with extensive experience in rhinoplasty. By rigorously evaluating these responses according to the criteria, we sought to establish the utility of AI-generated language models in simulating doctor-patient consultations.

### Inclusion Criteria

ChatGPT has a probabilistic algorithm and uses random sampling to generate varied responses, which can result in different answers to the same question. For this study, the first response provided by ChatGPT to each question was recorded, and subsequent clarifications or corrections were not allowed. Care was taken to ensure there were no grammatical or syntax errors in each question, and the questions were typed on the same day on a single ChatGPT Plus account owned by one of the authors. ChatGPT Plus is a subscription-only model of ChatGPT which purports to offer faster response times, priority access to new features, and access to the AI server even during periods of high demand. There is no publicly available information to determine if ChatGPT Plus utilizes a different algorithm to the standard model to generate responses.

## Results

**Fig. 1:**
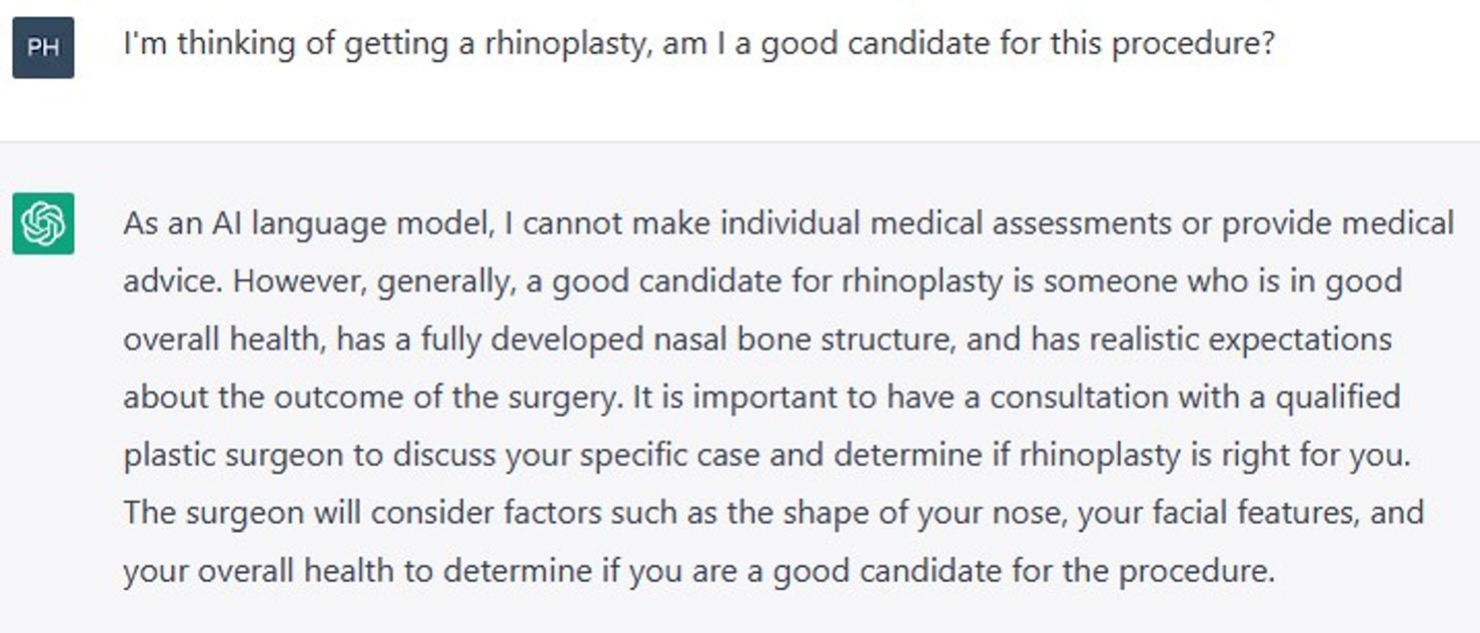
Prompt “I’m thinking of getting a rhinoplasty, am I a good candidate for this procedure?”

**Fig. 2:**
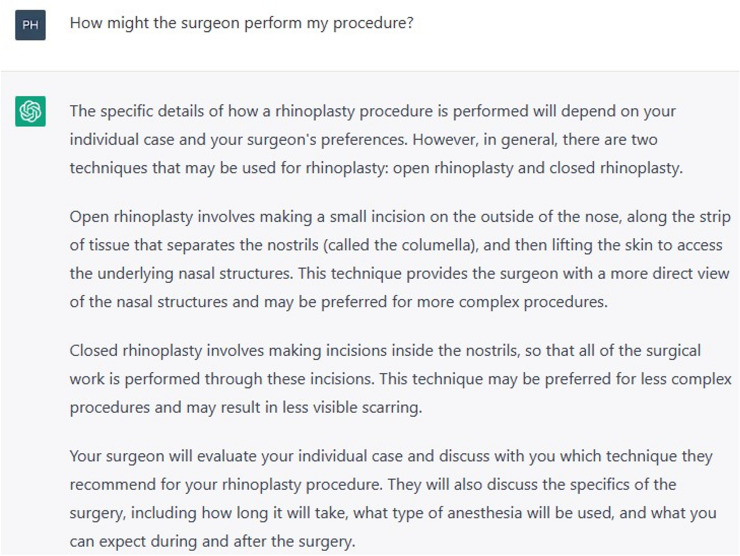
Prompt “How might the surgeon perform my procedure?”

To begin the consult, ChatGPT was asked “I’m thinking of getting a rhinoplasty, am I a good candidate for this procedure?” (Figure 1). This was followed up with “How might the surgeon perform my procedure?” (Figure 2).

ChatGPT prefaced its response to the first question with a disclaimer that it cannot make individual medical assessments or provide medical advice. It noted several general points that a patient should consider before undergoing a rhinoplasty: being in good overall health, having a fully developed nasal bone structure, and having realistic expectations about the outcome. Other relevant and important points such as patient objectives, cultural differences, and the cessation of smoking were not raised. ChatGPT outlined the two main approaches to rhinoplasty: open and closed, with only a perfunctory description of each.

**Fig. 3:**
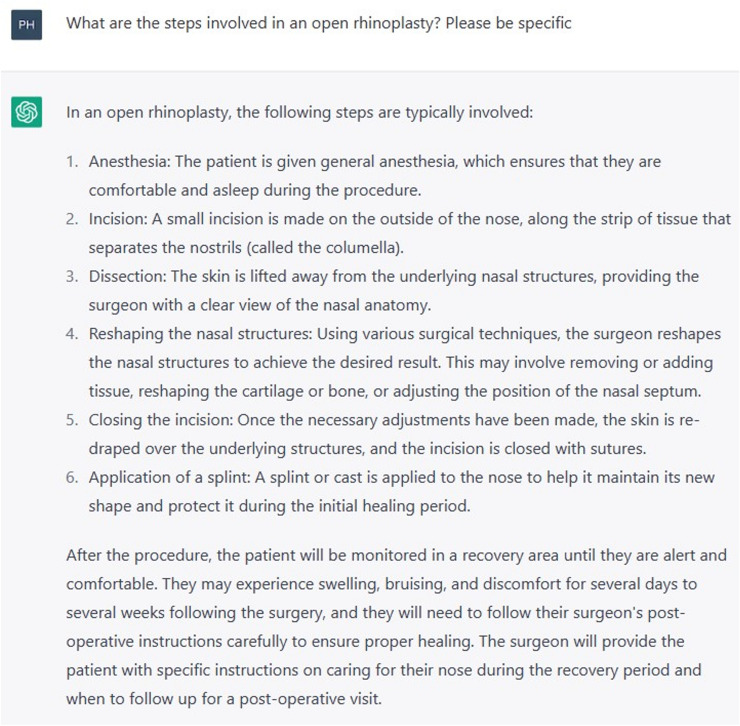
Prompt “What are the steps involved in an open rhinoplasty? Please be specific”

**Fig. 4:**
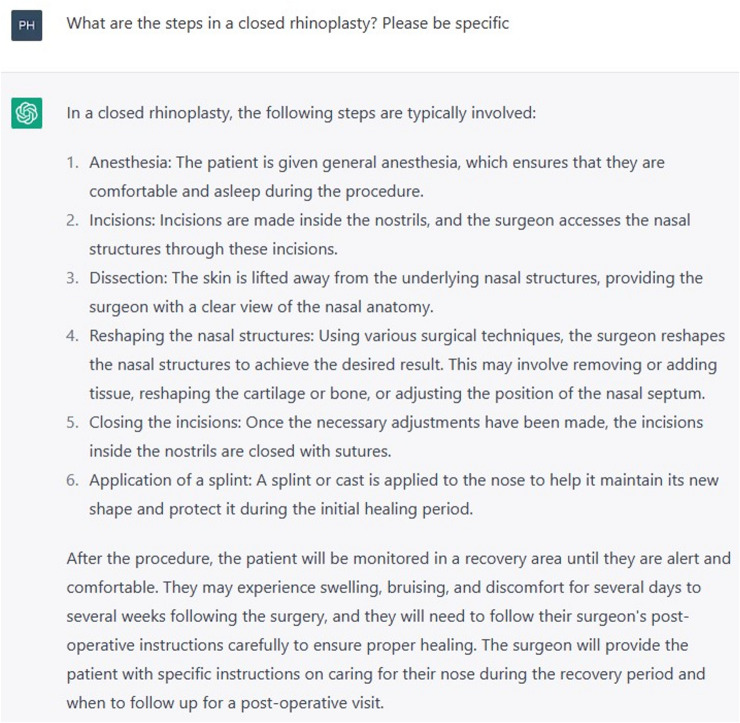
Prompt “What are the steps in a closed rhinoplasty? Please be specific”

**Fig. 5:**
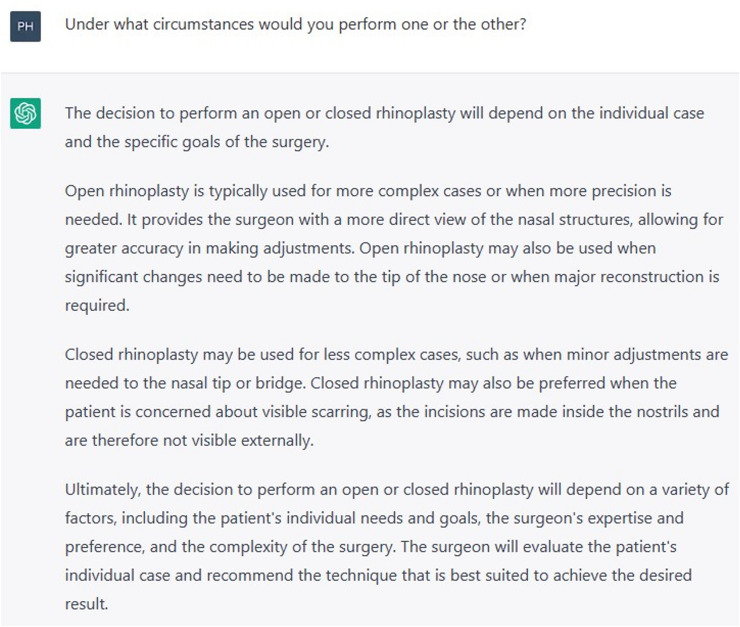
Prompt “Under what circumstances would you perform one or the other?”

ChatGPT was invited to elaborate on its response with three further prompts “What are the steps in a closed rhinoplasty? Please be specific” (Fig. 3), followed by “What are the steps in an open rhinoplasty? Please be specific” (Fig. 4). Thirdly, ChatGPT was prompted “Under what circumstances would you perform one or the other?” (Fig. 5).

Except for the incisional approach, ChatGPT gave the same response to both questions. Although the incision is an important distinction, ChatGPT failed to grasp the other technical differences between the two procedures. Its description of the dissection, for example, stated that “the skin is lifted away from the underlying nasal structures, providing the surgeon with a clear view of the nasal anatomy.” This generic statement inadequately portrays some of the challenges associated with the endonasal approach, such as the relatively limited surgical view, or the difficulties in the dissection of the alar cartilages, particularly if they are mispositioned. It also did not distinguish the difference in postoperative care for both procedures, which varies due to the often-decreased operative time and faster recovery for closed rhinoplasties. ChatGPT provided an adequate summary of when one might be preferable to the other, such as an open rhinoplasty for more precise correction of complex deformities, and a closed approach if the aim is to make conservative modifications and avoid external scarring [6]. Again, a disclaimer is provided, advising that the decision should be made with the surgeon on an individual basis.

**Fig. 6:**
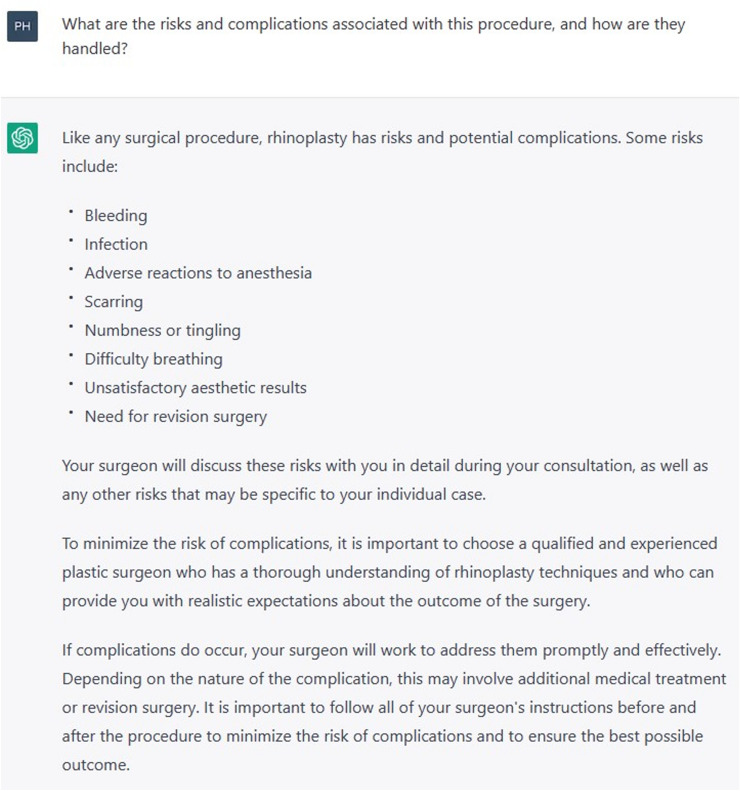
Prompt “What are the risks and complications associated with this procedure, and how are they handled?”

Following the procedural questions, ChatGPT was asked “What are the risks and complications associated with this procedure, and how are they handled?” (Figure 6). Informed consent before a surgical procedure plays a vital legal, ethical, and administrative role in patient care. It ensures that patients fully comprehend the procedure they are about to undertake and agree to accept the outcomes, including any complications that may arise during or as a result of the surgery. Recent studies have demonstrated that the addition of a digital information provision platform improves overall patient understanding, and satisfaction, and reduces consultation time for the surgeon conducting the consent discussion [7, 8]. ChatGPT responded with some complications common to all surgeries and some general complications of rhinoplasty. Risks associated with specific procedures, such as implants, or rare occurrences, such as orbital or endocranial complications, were not listed [9]. Given the impracticality of listing all the conceivable risks, ChatGPT correctly highlighted that the surgeon should be involved in a more in-depth discussion of each case.

**Fig. 7:**
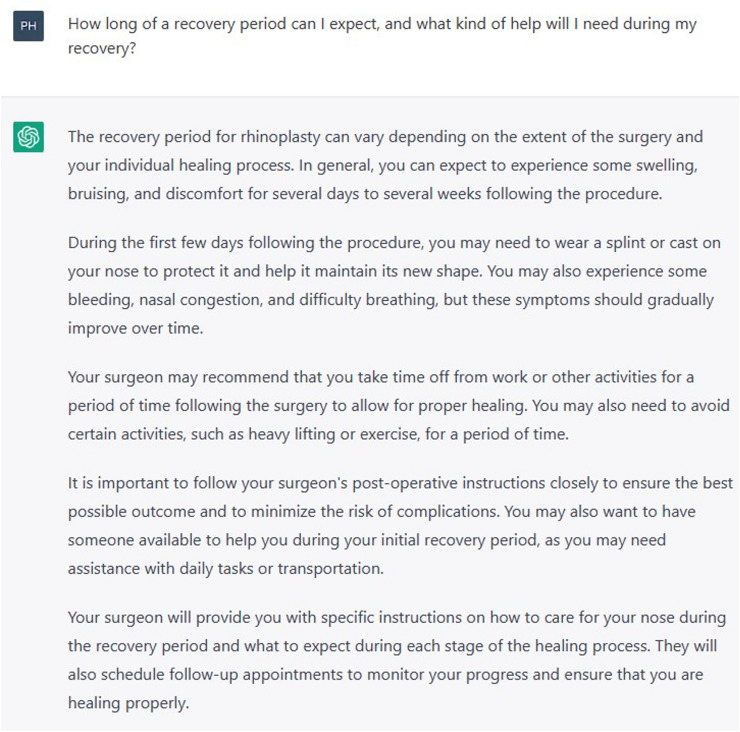
Prompt “How long of a recovery period can I expect, and what kind of help will I need during my recovery?”

**Fig. 8:**
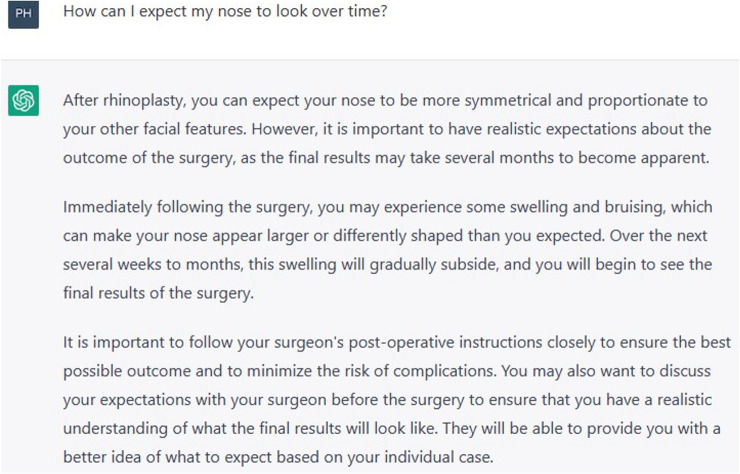
Prompt “How can I expect my nose to look over time?”

**Fig. 9:**
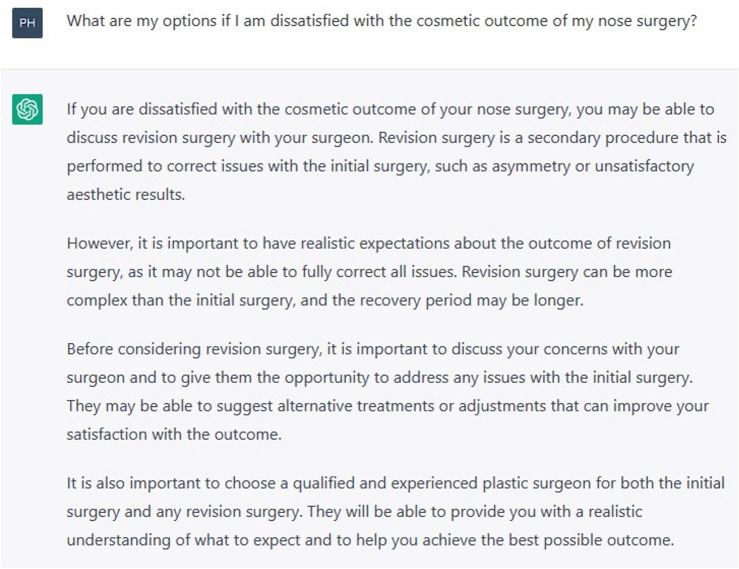
Prompt “What are my options if I am dissatisfied with the cosmetic outcome of my nose surgery?”

The rest of the consultation focused on the postoperative period. ChatGPT was queried “How long of a recovery period can I expect, and what kind of help will I need during my recovery?” (Figure 7). “How can I expect my nose to look over time?” (Figure 8). Finally, “What are my options if I am dissatisfied with the cosmetic outcome of my nose surgery?” (Figure 9).

ChatGPT gave a reasonable estimation of the recovery process, describing swelling and bruising for weeks to months, and advising the avoidance of certain activities. However, the discussion of aesthetic outcomes required a balanced and nuanced approach that we expected would be beyond its scope to emulate. There was an emphasis on having realistic expectations of the result, as well as multiple prompts to discuss the outcome and any dissatisfaction with the treating surgeon, including the availability of secondary procedures. Management of patients’ diverse operative, social and cultural expectations require empathy and rapport, elements of human interaction that cannot be obtained through text. ChatGPT recognized this limitation and appropriately directed the patient to follow the surgeon’s guidance in all regards during the operative and postoperative period.

## Discussion

This exploratory study demonstrates ChatGPT’s understanding of natural language in a health-specific context. It provided coherent answers that were easily comprehended and sufficiently informed. ChatGPT recognized its limitations in providing more esoteric advice, consistently cautioning the patient that each case ought to be evaluated by the surgeon and to follow the surgeon’s instructions pre- and postoperatively.

There is considerable debate in the scientific community regarding the implications of generative AI for science [10]. Machine learning and AI now have a big impact on most aspects of modern life and have seen increasing utilization in the medical field [11]. Deep learning systems have already shown diagnostic capabilities comparable to fully qualified specialists [12]. Before the reveal of ChatGPT in November 2022, IBM’s Watson was another system that used machine learning and natural language processing to analyse large datasets and provide insights [13]. IBM Watson received considerable public and media attention for its potential applications in cancer management, with treatment recommendations for some cancers demonstrating a level of concordance on par with oncologists in a specialist cancer centre [14]. ChatGPT has already demonstrated a passing performance equivalent to an undergraduate third-year medical school student on the US medical licensing exam [15]. Attempts to test ChatGPT in the clinical setting have resulted in the largely sound provision of antimicrobial advice, appropriate to the diagnosis [16].

The authors sought to explore the performance of ChatGPT in scenarios less reliant on algorithmic decision-making. With a predicted shortfall of 18 million health workers by 2030 as estimated by the World Health Organization (WHO), there is increasing concern for remote and rural populations which have traditionally struggled with healthcare worker retention [17]. Large language models (LLMs), the class of computer systems to which ChatGPT belongs, use deep learning algorithms to analyse language patterns and predict the sequence of words most likely to follow in a sentence or text passage [18]. With training from diverse internet text sources, ChatGPT can respond to a wide range of topics. There is value in exploring the application of AI models such as ChatGPT in the emerging space of digital clinical guidance. For example, by leveraging advancements in AI language processing with comprehensive medical databases, there is potential to design chatbots capable of providing effective and safe, if generalized, medical advice to patients.

Patient selection and the setting of realistic expectations are two of the key factors in the consultation pathway for aesthetic surgery. This is of particular importance in rhinoplasty, simultaneously one of the most sought after, and most complex procedures in Plastic Surgery [19]. The link between rhinoplasty, psychology, and social environment is well established in the literature, with emphasis placed on the importance of patient selection to avoid not only physical but psychological postoperative complications [20]. While various tools, including a body dysmorphic disorder questionnaire, have been proposed to assist in patient selection [21], there is no consensus instrument to predict poor outcomes or patient dissatisfaction postoperatively. Accordingly, while AI has enormous potential for improving healthcare in the areas that rely on algorithmic decision-making, such as diagnostics, resource allocation, and data collection, its application is currently limited in clinical settings where empathy and compassion are paramount.

In the context of rhinoplasty, ChatGPT can serve as a valuable resource for patients seeking information about the procedure, its risks, benefits, and outcomes. Patients can ask questions in natural language and receive immediate responses, which can help to clarify any misunderstandings and set some expectations. ChatGPT can also provide patients with information about the surgeon’s credentials and experience and help them to make informed decisions about their care. Additionally, ChatGPT can assist surgeons by providing them with information about the patient’s medical history and current health status, which can help them in the preoperative assessment and development of an appropriate operative plan. While ChatGPT cannot replace the psychological competency and empathy of a surgeon, it can serve as a valuable adjunct to the consultation process.

## Conclusions

This exploratory study provides insights into the utility of AI-generated language models in simulating doctor-patient consultations for rhinoplasty. ChatGPT demonstrated an understanding of natural language in a health-specific context and provided coherent, information, and accessible answers. While it recognized its limitations in providing esoteric and personal advice, it was able to assist patients with basic information about the procedure, its risks, benefits, and outcomes. ChatGPT can be a valuable resource for patients seeking information and surgeons in preoperative assessment and planning. However, patient selection and setting realistic expectations remain essential factors in the consultation pathway for aesthetic surgery, an area in which the role of current-generation AI remains limited. Further research is needed to explore the potential of AI models such as ChatGPT in digital clinical guidance and the broader healthcare context.
